# The Effects of Extreme Weather on Apple Quality

**DOI:** 10.1038/s41598-020-64806-7

**Published:** 2020-05-13

**Authors:** Tobias Dalhaus, Wolfram Schlenker, Michael M. Blanke, Esther Bravin, Robert Finger

**Affiliations:** 1 Agricultural Economic and Policy Group, ETH Zürich, Zürich, Switzerland; 2 School of International and Public Affairs & the Earth Institute, Columbia University, New York, USA; 3 INRES Horticultural Science, University of Bonn, Bonn, Germany; 4 Competence Division for Research Technology and Knowledge Exchange Plants and Plant Products, Agroscope, Zürich, Switzerland

**Keywords:** Plant sciences, Natural hazards, Environmental economics

## Abstract

A large literature has documented the effects of weather on agricultural yields. However, weather not only impacts the quantity produced, but also the quality of the product. Due to data limitations, the quality effects have primarily been studied using lab experiments for specific attributes, and the financial implications for farmers of a quality effect are less clear. Using a unique longitudinal micro-level data set of Swiss apple orchards that include information on both the quantity produced as well as the quality, we show that the latter can have an even larger effect on farm revenue. Ignoring the quality of the harvested product substantially biases the impact of weather extremes on agricultural income and the potential effects of climate change. Our quality measure is the orchard-year specific price shock. If an orchard gets a lower price for its specific apple variety compared to previous years and compared to other orchards in the same year, we observe the market’s valuation of its inferior quality accounting for overall price movements (other orchards growing same variety that year) as well as orchard specific factors (orchard fixed effects). We find that spring frost events induce farm gate price drops and thus revenue reductions of up to 2.05% per hour of exposure.

## Introduction

Weather impacts both crop yield quantity and quality and is thus a driving force of farm income volatility^1^. While yield quantity risks and their determinants are usually well documented, yield quality risks are widely neglected. Possible effects of weather on parameters that impact the quality of a crop are, for example, the outer appearance^2–4^, specific valuable constituents^5,6^ or the textural structure^7^ of the harvest. So far, the market valuation of these effects remains unquantified. What ultimately matters for farmers’ incomes is how changes in the quality of products translate into price changes. Ignoring the important channel on how weather extremes translate into income losses due to a change in quality (and associated prices) potentially biases the impact of extreme weather events and climate change on agricultural production.

We use orchard and year-specific price shocks to assess how weather impacts apple quality and ultimately farm revenue. Our unique data set gives prices for orchards that each grow one particular apple variety over several years. This allows us to construct a causal link between weather and the quality-induced income effect. Intuitively, say both orchards A and orchards B grow Golden Delicious apples for several years. The average prices the owners of each orchard receive might differ due to differences in average quality or simply because one has better business acumen, e.g., superior supply chain management arrangements. We therefore look at the evolution of price differentials over time. If orchard A gets on average 5 cents more per kg of apple, but this difference narrows to 2 cents in a given year, we attribute the 3 cents price decline to the observed weather the orchard faces. In technical terms, the orchard fixed effects account for time-invariant differences of each orchard and by differencing with other orchards through the inclusion of year-fixed effects we account for market wide price movements. Since weather is random and exogenous, it should not be correlated with other time varying factors and hence the resulting change in price differentials can be causally attributed to the observed weather fluctuation. We estimate how much deviations from optimum quality traits translate into drops in realized producer prices. By doing so we are able to disentangle the overall effect of weather on revenues into its yield and price components. While the quality channel has been mentioned before^8^, we offer a novel approach to estimate it empirically.

Amid lacking site-specific realized price information, past literature has been unable to quantify the economic effect of weather extremes on harvest quality, while the agronomic mechanisms how weather can negatively influence crop quality are only known from field experiments [e.g.^9–11^].

**Figure 1:**
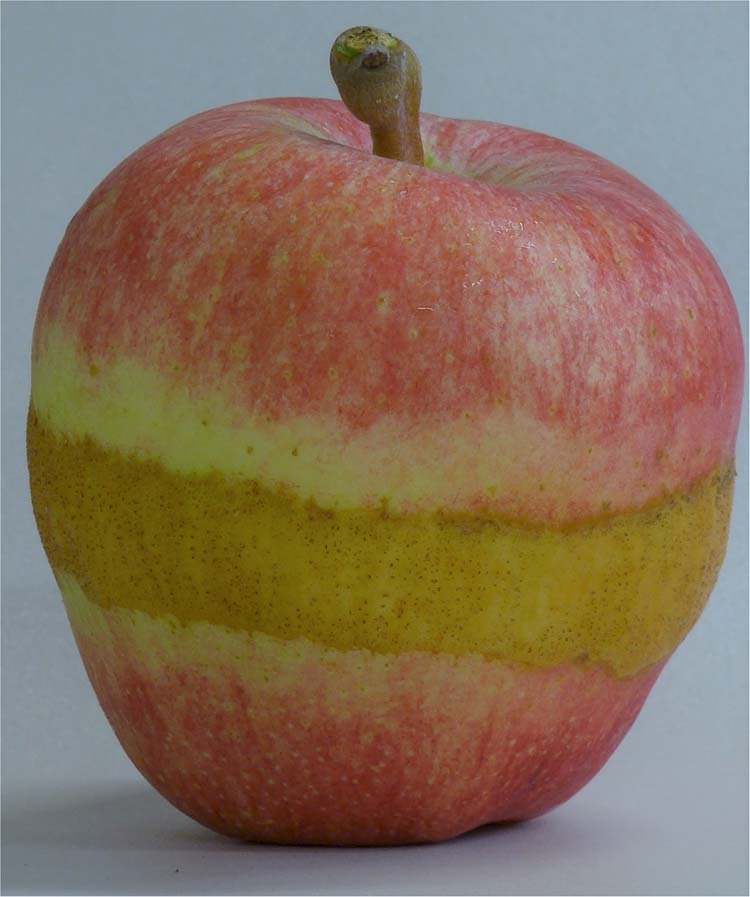
Morphologically damaged apple caused by spring frost events during flowering that partly damaged the blossoms, which resulted in a “Frost Ring”. The image was provided by Door Creek Orchard, Cottage Grove, WI, USA.

Our data consists of a unique orchard-level panel dataset on apple yield and price records consisting of 2′462 observations from 1997 to 2014 in Switzerland. This allows us to investigate how late spring frost events impact apple quality. Spring frost events are known to cause morphologically damaged apples (see Fig. 1 and supplementary section S1 for an overview on plant physiological mechanisms). We match orchard-level price and yield observations with regional tree phenology and historical daily temperature records. We use a piecewise linear regression approach proposed by Schlenker & Roberts^12^ that puts a special focus on extreme temperature events, which in our case turn out to be low temperature events. Furthermore, our model controls for other temperature impacts besides spring frost on apple trees, such as beneficial winter chilling and summer heat, as these might be correlated with spring frost. We use plot level fixed effects to control for time invariant single orchards characteristics, such as variety, production system, soil conditions. To control for overall market movements in prices we use year dummies.

Our results show that idiosyncratic spring frost events induce only minor drops in yields while they cause farm gate price and thus revenue reductions of up to 2.05% per hour of exposure. Our findings highlight that frost effects on crop yield quality clearly outweigh effects on quantity. Never being quantified in any region for any crop before, these findings on weather induced quality reductions are of particular relevance for agricultural risk and risk management literature but also add to the climate change impacts literature, as late frosts are more likely to occur in the future and effects of climate change on crop quality have received limited attention so far^9,13,14^.

## Results

**Figure 2:**
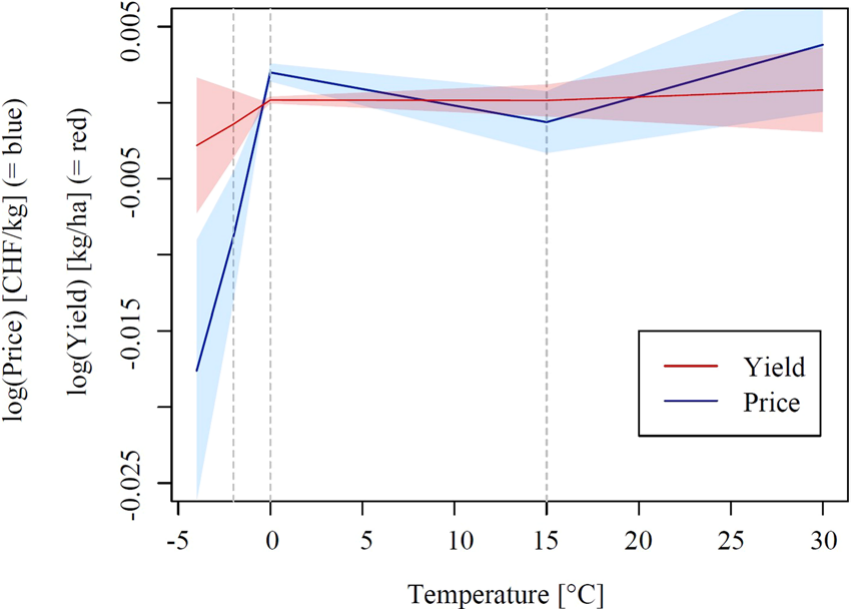
Non-linear price (blue) and yield (red) response to a one-hour temperature exposure at the respective x-axis temperature during apple flowering. Shaded areas represent 95% confidence bands when errors are clustered by year and orchard. Dashed grey lines indicate interval breaks of the piecewise linear specification of the temperature impact.

We empirically estimate the influence of temperatures on both apple quantity and quality. The results are shown in Fig. 2. The piecewise linear function is akin to the concept of degree days, which measure how much and how long temperatures exceed or fall below a certain threshold. For example, degree days below 0 °C measure how long and by how much temperatures fall below the threshold. Being 4 hours at −1 °C or two hours at −2 °C would both result in 4 degree days below 0 °C. Figure 2 shows temperature impacts across different temperature intervals. We find the impact of temperature exposure during flowering on prices (blue line) being highly nonlinear with large drops under freezing conditions. Here, idiosyncratic farm gate prices start dropping at slightly below 0 °C. This effect increases up to a loss of 1.76% when the apple orchard is exposed for one hour to −4 °C (See appendix Figure S1 for revenue drops of −2.05% for this temperature). As market (supply and demand) induced price shifts are controlled for by year dummies, this effect is idiosyncratic, only affecting single orchards. We thus conclude that this is determined by a frost induced downgrading of a certain share of the apple harvest. This share is increasing with decreasing temperatures. On the other hand, the red line in Fig. 2 shows that there is no similar relationship for the apple quantity produced (See Table S2 for full regression results and Figure S9 summaries on the temperature exposure across different temperatures). In summary, quality losses induced by weather extremes substantially outweigh quantity losses in our case study example of spring frost in apple production. In fact, for moderate spring frosts during apple flowering we find a negative but not statistically significant effect on yields while realized selling prices substantially drop.

Supplementary Figures S2 and S3 show that our findings are robust to changes in the temperature interval choice. We also experimented with flexible cubic splines that do not impose a linear relationship, but the estimated relationship at the extremes closely mirrors a linear relationship.

## Discussion

Many studies estimated the impact of temperature extremes on crop yields and the resulting monetary implications for farmers^12,15,16^. While this helped to better understand how weather extremes impact volumetric crop yield losses, farmers’ revenues are also substantially driven by the realized selling price of the harvest, which itself depends on the quality grade of the final product^8,17^. Multiple examples on weather induced quality losses exist, however, proxies to quantify the impact of weather on quality economically are missing^5,9–11^. Our understanding of how weather extremes impact agricultural production is incomplete.

Our findings fill this gap by proposing that the realized site-specific selling price deviations can serve as proxy for the quality of the harvest, and the price effect of the quality changes is what farmers are ultimately interested in. We see it as a strength that we are relying on price data instead of measurements of an apple’s quality without knowing how the market ultimately prices such quality differences. The main empirical weather condition in our setting of apple production that impacts an apples quality is spring frost. In this instance, economic consequences of quality losses can outweigh those on quantity substantially. Our empirical procedure allows to identify this effect for an orchard growing a specific variety across years by linking it to random weather variation, which are idiosyncratic and random. The mechanism are morphological damages such as frost rings (Fig. 1) but can also be caused by pests that are supported by the frost events that might manifest in rotten fruits^18^.

Supplementary Figures S4–S8 estimate the relationship for particular apple varieties. In a placebo test we find that the price of processing varieties for which the outer appearance is of minor important, such as *Boskoop*, does not respond to frost exposure as expected, highlighting the validity of our assumption that frost is uncorrelated with other factors. We observe the market’s response to a reduction in apple price only for varieties that have shown to be impacted by the apple frost in this way. The novelty of our study is to monetize this effect, and measure the income implications for farmers. Given the limited number of observations for some apple varieties, the error bands tend to widen if we limit the data to a particular variety and the coefficient estimates are mostly not significantly different from the average effect.

Leaving this important mechanism unconsidered could dramatically underestimate the impact of extreme events on agricultural production and could bias farmers’ expected response to climate change. Simulation studies that predict farmers’ response to climate change should consider all climate change induced alterations in the risk exposure and its monetary consequences to avoid biased predictions^19,20^. Indeed, focusing on yield losses only, ignoring realized prices as important part of farmers’ revenues, which constitutes farmers’ most important goal variable, makes us unable to simulate farmers’ behavior under climate change conditions.

We estimate a reduced form model that does make assumptions on the relationship between weather and outcome variables^21^. Thus, the apple producers might apply agro-managerial practices and our estimated relationship allows for these short-term adaptations^22^. The resulting effect can be interpreted as the spring frost response of yields and prices after short-term adaptation. Moreover, production costs might be affected by spring frost events, which itself impacts overall profits, but our dataset does not include costs at the plot level. Our estimates are hence a lower bound on the detrimental effects of spring frost, as they include the beneficial effects of short-term adaptation, but exclude the cost of such measures. We see this as an interesting topic for future research. Note that weather induced costs as well as the weather induced yield quantity and quality losses can, for example, be reduced by designing appropriate weather index insurance solutions. Such insurance would pay out to farmers in a frost event, independently of the physical damage. Thus, producers can receive an insurance payout even without facing yield losses due to the use of preventive, but costly, measures^23–25^.

We here use heat stress (number of day >30 °C) as a control variable. When being particularly interested in the impact of extreme heat, it can be modelled more flexibly, e.g. with a non-linear specification such as regressions splines, the piecewise linear approach used here for the frost impact or polynomial specifications (see Blanc & Schlenker^22^ for an overview). As we do neither expect nor find any correlation between spring frost and summer heat events we decided for this more straightforward summer heat control variable. It should be noted that simple statistical models outperformed biophysical models in an out-of sample prediction exercise where various modeling groups predicted yields for real world test plots before knowing their yields^26^.

Important for apple trees, climate change is expected to cause increases in winter temperatures leading to changes in the fulfillment of cooling (chilling) and heat requirements (forcing) that induce the end of the apple trees’ winter dormancy^13,27–30^. Thus, apple blooming is more likely to be affected by spring frost events across temperature regimes analyzed here, constituting an increase in the downside risk exposure of apple producers^31–35^.

A complete picture of extreme weather impacts on agricultural production is a crucial prerequisite for measuring the full effects of climate change. So far, weather induced crop quality losses are largely ignored although agronomic knowledge suggests strong relationships. Neglecting these quality losses that likely occur also in other crops, downward biases our understanding of the impact of weather extremes on agricultural incomes and therefore threatens food security. Future research should estimate the impact of weather events on quality also beyond our apple example, when site specific price data becomes available.

Many policy instruments (extension, education, financial support, subsidies to insurance premiums) are targeted on the reduction of farmers’ financial risk exposure, but often ignore the role of crop quality losses. Thus, our results deliver important insights for policy makers to design better polices that aim at supporting farmers in low income situations.

## Empirical Methods

We use regression analysis to estimate the impact of temperature exposure during apple flowering on apple price ${p}_{it}$ and yield ${y}_{it}$ at orchard *i* in year *t*. Therefore, we estimate the following models:

1 $$\log \,({p}_{it})=f({T}_{ti},\beta )+\delta {z}_{it}\,{+}_{i}{+}_{t}+{\varepsilon }_{it}$$

2 $$\log \,({y}_{it})=g({T}_{ti},b)+d{z}_{it}\,+{a}_{i}+{c}_{t}+{e}_{it}$$

where *log* (*p*_*it*_) and *log* (*Y*_*it*_) are the natural logarithms of the apple price [CHF/kg] and yield [kg/ha] of orchard *i* in year *t* that grows a specific apple variety^36^. The function $f({T}_{ti},\beta )$ and $g({T}_{it},b)$ allow for the potentially non-linear effects of temperature exposure during various time periods of the growing season, e.g., flowering. The controls ${z}_{it}$ denote an orchard and year specific matrix of temperature variables that have previously been shown to impact apple output, i.e., chilling hours in the winter and heat spells in the summer. All time invariant unobserved heterogeneity is controlled for in the orchard-level fixed effects *i* and ${a}_{i}$. Hence, our estimates are not affected by orchard characteristics that lead to shifts in average orchard price or yield levels (such as variety, production method (i.e. organic), surface texture or microclimate). In our sample, observations are structured in a way that only one variety is grown on a single orchard. We are thus able to explicitly control for variety effects. Moreover, see appendix Figures S4–S8 for subsample results for the five most common varieties. We include y and ${c}_{t}\,$to capture all systemic price and yield movements that occur across all orchards jointly in each year. Consequently, we model the impact of temperature exposure on yields and prices when these deviate from their long-term orchard average and from the other observations in a given year (i.e. systemic price movements e.g. due to policy changes or market effects and systemic yield movements through pestoutbreaks in the entire country). The estimated impact is thus idiosyncratic. The error terms ${\varepsilon }_{it}$ and ${e}_{it}$ are likely heteroskedastic, spatially correlated within each year, and temporally correlated within one orchard over the years. We therefore use heteroscedasticity robust standard errors that are two-way clustered by year and orchard^37^. We follow Schlenker and Roberts^12^ and use a piecewise linear approach to model $f({T}_{ti},\beta )$ and $g({T}_{it},b)$ Therefore the temperature impacts on prices and yields during the flowering phase are modelled as:

3 $$f({T}_{ti},\beta )={\beta }_{1}{T}_{(-\propto ,-2]{}^{^\circ }C}+{\beta }_{2}{T}_{[-2,0]{}^{\circ }C}+{\beta }_{3}{T}_{[0,15]{}^{\circ }C}+{\beta }_{4}{T}_{[15,){}^{\circ }C}$$

4 $$g({T}_{ti},b)={b}_{1}{T}_{(-\propto ,-2]{}^{\circ }C}+{b}_{2}{T}_{[-2,0]{}^{\circ }C}+{b}_{3}{T}_{[0,15]{}^{\circ }C}+{b}_{4}{T}_{[15,){}^{\circ }C}$$

In (3), ${T}_{(-\propto ,-2]{}^{\circ }C}$ measures by how much and for how long temperatures fall below −2 °C, the same concept underlying degree days that are often used as factors driving agricultural output. For example, being 4 hours at $-3^\circ C$ would result in an exposure of 4, as would being one hour at $-6\,^\circ C$. Temperature intervals with two bounds, e.g., ${T}_{[-2,0]^\circ C}$,measure by how much and for how long temperatures exceed the lower bound (−2 °C), while temperatures are truncated at the upper bound (0 °C), i.e., any temperature above 0 °C will be recorded as two (difference between upper and lower bound for the interval in question). The analogous definition applies for the temperature interval ${T}_{0^\circ C-15^\circ C}$. Finally, ${T}_{[15,\propto )^\circ C}$ measures for how long and by how much temperatures exceed 15 °C. The motivation of these temperature intervals come from a more flexible regression using cubic splines (third-order approximations) that exhibited clear strong nonlinearities at both ends of the temperature distribution. (See Supplementary Figures S2 and S3 for results using different interval specifications and section S1 for background information on the plant physiological importance to distinguish between different freezing temperatures). For impacts of temperature exposure on overall revenues (price multiplied by yield) see appendix Figure S1.

## Data

### Orchard data

Economic orchard-level panel data on apple production were provided by the Swiss Federal research station Agroscope^38^. This dataset is unique in capturing site specific realized price information. We are not aware of a comparable data set that gives the same fine-scaled (orchard-level) market assessment of apple quality. The unbalanced dataset includes 2′462 observations containing information on 55 apple varieties planted on 505 orchards across ten cantons (Swiss Federal States) during the years 1997–2014. Observed variables include yields (2′444 observations) [kilogram (kg)/ hectare (ha)], revenues (2′389 observations) [CHF (Swiss Francs)/ha] (1 CHF = 0.93 EUR), farm-gate prices (2′389 observations) [CHF/kg], variety, municipality and a dummy on whether the orchard produces organic (See Table S1 for descriptive statistics). For 18 observations we have no information on either yields, prices or revenues. Thus, these 18 observations are dropped from the further analysis. The farm-gate prices are orchard specific average realized prices. Thus, a larger share of downgraded apples leads to lower prices. Most common varieties in our sample were Golden Delicious (16% of all orchards), Gala (10%) and Jonagold (8%). We apply a multivariate outlier detection procedure to identify and remove outliers in our data using the bacon algorithm based on Mahalanobis distances^39^. We thus removed four outlying price and three yield observations. For the respective variables our final dataset thus results in 2′441 observations for yields and 2′385 for prices respectively, which we include in our analysis. For further information on the orchard data see the supplementary section *S2 Economic orchard data*.

### Apple tree phenology data

Start and end dates of the apple blooming period (i.e. from growth stages BBCH 61 *“first flowers open”* to BBCH 69 *“End of flowering: all petals fallen”*^40^) in each orchard, year and variety were found using phenological records of experimental sites across our study region. More specifically, we obtain our tree phenology data from 33 different experimental stations across Switzerland. The dataset includes location information of the experimental station and occurrence dates of growth stages along the BBCH scale. The data can be accessed via an online form at http://www.agrometeo.ch. We use phenology information to find start and end dates of flowering (BBCH 61 to BBCH 69) and chilling (BBCH 97 and BBCH 00; see section on weather data for further information on temperature control variables)^41^. Dependent on data availability we consider the following four steps of assigning phenology data to single orchards^36^.

- First we match the single orchard information with variety specific phenology data within the same year and canton (here we find 1,176 matches). We then use the earliest/latest date within BBCH 61 and BBCH 69 to find the start/end date of flowering for the single orchard.
- Second, in case of insufficient data for step one, we match orchard information with all available variety phenology (except the early flowering variety Boskoop) within the same year and canton (here we find 1′020 further matches). We then use the earliest/latest date within BBCH 61 and BBCH 69 to find the start/end date of flowering for the single orchard.
- Third, in case of insufficient data for step two, we match orchard information with variety specific phenology within the same year in all cantons (here we find 141 further matches). We then use the earliest/latest date within BBCH 61 and BBCH 69 to find the start/end date of flowering for the single orchard.
- Fourth, in case of insufficient data for step three, we match orchard information with phenology information within the same year across all varieties from all cantons (here we find 107 further matches). We then use the earliest/latest date within BBCH 61 and BBCH 69 to find the start/end date of flowering for the single orchard.

See Figure S11 for a histogram of start and end dates of flowering. Our results are robust against dropping the 248 observations from the last two nationwide matching steps (See Supplementary Figure S9).

### Weather data

Daily minimum and maximum temperatures within tree phenology stages were obtained from a gridded (raster) dataset with 2.5 ×2.5 km resolution provided by the Swiss Meteorological Office^42^. The interpolation method to produce the temperature raster data specifically considers Swiss specific texture characteristics and thus nonlinear temperature changes across elevation levels. Furthermore, “valley-scale cold-air pools” can be realistically displayed, taking into account site specific micro climates^36^.

To obtain time spend in the different temperature intervals described earlier, we use the procedure proposed by^43^. We fit a sine curve between daily maximum and minimum temperature and derive the daily exposure spent in each temperature interval. The sum of all daily exposures during the flowering period across the intervals defined in the methods section is then used in the regression analysis. See Figure S10 for boxplots of the temperature exposure [in days per degree].

We further use a set of variables to control for other than frost damage effects of temperature. More specifically, we control for damaging summer heat and beneficial winter chill. Regarding summer heat, we use the number of days with a maximum air temperature above 30 °C to control for summer heat spells that potentially damage apple fruits (see Racsko & Schrader^44^ for an overview). Regarding winter chill, we control for beneficial cooling effects by including a winter chill control variable. Here we use the available chilling hours (hours with air temperature between 0 °C and 7.2 °C) between leaf fall and bud development (BBCH 97 and BBCH 00) obtained from the phenology data, according to above matching procedure. For the chilling variable we use a similar procedure as for the temperature during flowering variable. We estimate a sine curve between the daily maximum and minimum temperature variables to derive hourly temperatures^43^. From that we derive the exposure time between 0 °C and 7.2 °C.

## Supplementary information

Supplementary Information.

## Data Availability

The underlying data are available from the Swiss Federal research station Agroscope but restrictions apply to the availability of these data, which were used under license for the current study, and so are not publicly available. Data are however available from the authors upon reasonable request and with permission of the Swiss Federal research station Agroscope.
