# Publisher Correction: The Effects of Extreme Weather on Apple Quality

**DOI:** 10.1038/s41598-020-69048-1

**Published:** 2020-07-17

**Authors:** Tobias Dalhaus, Wolfram Schlenker, Michael M. Blanke, Esther Bravin, Robert Finger

**Affiliations:** 1 Agricultural Economic and Policy Group, ETH Zürich, Zürich, Switzerland; 2 School of International and Public Affairs & the Earth Institute, Columbia University, New York, USA; 3 INRES Horticultural Science, University of Bonn, Bonn, Germany; 4 Competence Division for Research Technology and Knowledge Exchange Plants and Plant Products, Agroscope, Zürich, Switzerland

Correction to: *Scientific Reports* https://doi.org/10.1038/s41598-020-64806-7, published online 13 May 2020

This Article contains errors in Equation 1.

$$ \log (p_{it} ) = f\left( {T_{ti} ,\beta } \right) + \delta z_{it} + _{i} + _{t} + \varepsilon_{it} $$

should read:

$$ \log (p_{it} ) = f\left( {T_{ti} ,\beta } \right) + \delta z_{it} + \alpha_{i} + \gamma_{t} + \varepsilon_{it} $$

Additionally, there are typographical errors in the Empirical Methods section where,

“All time invariant unobserved heterogeneity is controlled for in the orchard-level fixed effects $i$ and $a_{i}$”

should read:

“All time invariant unobserved heterogeneity is controlled for in the orchard-level fixed effects $\alpha_{i}$ and $a_{i}$”

and,

“We include y and *c*_*t*_ to capture all systemic price and yield movements that occur across all orchards jointly in each year.”

should read:

“We include $\gamma_{t}$ and $c_{t}$ to capture all systemic price and yield movements that occur across all orchards jointly in each year.”

