# Supplementary material for: The Effects of Extreme Weather on Apple Quality
Source: Sci Rep. 2020 May 13;10:7919. doi: 10.1038/s41598-020-64806-7 (PMC7220945; doi:10.1038/s41598-020-64806-7)
Supplement: Supplementary file 1 — Supplementary Information. [file 41598_2020_64806_MOESM1_ESM.docx]

Supplementary Information

The Effects of Extreme Weather on Apple Quality

Tobias Dalhaus^1*^, Wolfram Schlenker^2^, Michael M. Blanke^3^, Esther Bravin^4^, Robert Finger^1^

^1^Agricultural Economic and Policy Group, ETH Zürich, Switzerland

^2^School of International and Public Affairs & the Earth Institute, Columbia University, USA

^3^INRES Horticultural Science, University of Bonn, Germany

^4^Competence Division for Research Technology and Knowledge Exchange Plants and Plant Products, Agroscope, Switzerland

*corresponding author: [tdalhaus@ethz.ch](mailto:tdalhaus@ethz.ch)

Content

[S1 Background 2](#_Toc37145259)

[S2 Economic orchard data 3](#_Toc37145260)

[S3 Adaptation 5](#_Toc37145261)

[S4 Supplementary results 6](#_Toc37145262)

[References 18](#_Toc37145263)

## S1 Background

Our case study example focuses on the impact of spring frost in apple production. Apples are one of the most consumed fruits worldwide and late spring frost is well recognized as one of the greatest weather risks that can destroy large parts of the apple production (e.g. large parts of European apple production have been destroyed by heavy spring frost in the 2017 season) [38,18,14].Within the floral organs of apple blossoms, ice crystals start formation below a temperature of 0°C. The crystals grow from the extra cellular space, through the cell membrane into the reproductive organs’ cells, potentially causing multiple cell deaths. Depending on the severity of frost exposure, the affected flowers either continue to develop normally, produce anatomical and morphological damaged apple fruits or fully abort their development. These damages may cause effects to be observed in i) size, ii) appearance or iii) shape of the ripening fruit [14,25,26]. Volumetric yield effects of spring frost on apple yields and expected changes in the exposure due to climate change are well documented in the agronomic literature [18,25,26,27,28,39,40]. In contrast spring frost effects on fruit quality have not been quantified. This research gap is especially due to the fact that the complexity of quality aspects, restricts the straightforward quantification of impacts on apple fruit quality. However, apple quality is an important driver of economic success and quality induced losses may be more relevant than fluctuations in production quantities [41]. Thus, the assessment of climatic risks should be based on final aggregated economic effects that are crucial from the farmers’ perspective [42].

## S2 Economic orchard data

The investigated farms are located in the Swiss lowland and pre-alpine region, constituting the major apple growing region in Switzerland [43]. Switzerland’s apple producing regions are characterized by i) small scale farms and orchards, ii) rainfed fruit production in a temperate climate and iii) limited risk management options. More specifically, sprinklers, heating/fogging or wind machines, are usually not used yet due to limited economic or technical viability. We thus expect frost related losses in apple production to have a considerably negative influence on revenues while constituting one of the greatest climate related risks. An insurance for frost damages in fruit production was only introduced in Switzerland in 2018.

The farm level data is collected within an agricultural extension service network that aims at collecting representative economic information of growers to support advisory service and policy makers. Partner organizations are the Swiss Federal research station Agroscope ([www.agroscope.admin.ch](http://www.agroscope.admin.ch)), the agricultural extension service provider agridea (www.agridea.ch) and the Swiss fruit producer organization ([www.swissfruit.ch](http://www.swissfruit.ch)). Further information (in French and German) about the network can be obtained via [www.supportobstarbo.ch](http://www.supportobstarbo.ch). As the data contains individual economic data, access is restricted and can be requested at the Swiss Federal research station Agroscope.

The Swiss market is highly protected and characterized by import restrictions on many fresh fruits and vegetables. Thus, changes in domestic supply, e.g. through systemic weather extremes, likely induce changes in domestic producer prices [44]. Our econometric model explicitly controls for such market effects by using year fixed effects.

Although our dataset is not a randomized drawn sample of the Swiss apple grower population, it well represents the most common varieties and apple producing regions^[[1]](#footnote-1)^. Furthermore, the availability of single orchard price information makes the dataset in its form unique and justifies its usage in our analysis. We strongly recommend to capture single plot price data also for other crops to be able to capture revenue losses more precisely in future research.

Table S1: Descriptive Statistics of Economic Orchard Data

|  | **Mean** | **Minimum** | **Maximum** | **Standard Deviation** | **N** |
| --- | --- | --- | --- | --- | --- |
| **Yield [kg/ha]** | 32’397 | 1’125 | 88’408 | 16’332 | 2’444 |
| **Revenues [CHF/ha]** | 28’499 | 1.13 | 145’271 | 18’265 | 2’389 |
| **Farm gate price [CHF/kg]** | 0.95 | 0.01 | 4.77 | 0.49 | 2’389 |
| **Organic [dummy]** | 0.19 | 0 | 1 | 0.40 | 2’444 |

## S3 Adaptation

In order to cope with increasing frost risk, adaptation measures are needed. Although not relevant/possible in our case study context, some measures are widely be applied, which we explain here. If irrigation infrastructure is established, frost irrigation through sprinklers can be an efficient way of managing the impact of frost [45]. However, resource efficient water use is on the policy agenda of many countries, leading to limited opportunities to apply those sprinklers [46]. Other solutions, such as heating or wind machines are characterized by a high energy use [47]. Thus, more sustainable strategies to adapt to increasing spring frost risk need to be developed. To this end, agricultural insurances can be designed to help farmers to overcome frost related periods of illiquidity and the here presented study can contribute to develop better insurance mechanisms. Weather index insurances are straightforward solutions to cover weather related losses within specific phases of plant growth. Here, frost exposure within a critical crop growth phase triggers payouts to farmers [48,49].

## S4 Supplementary results


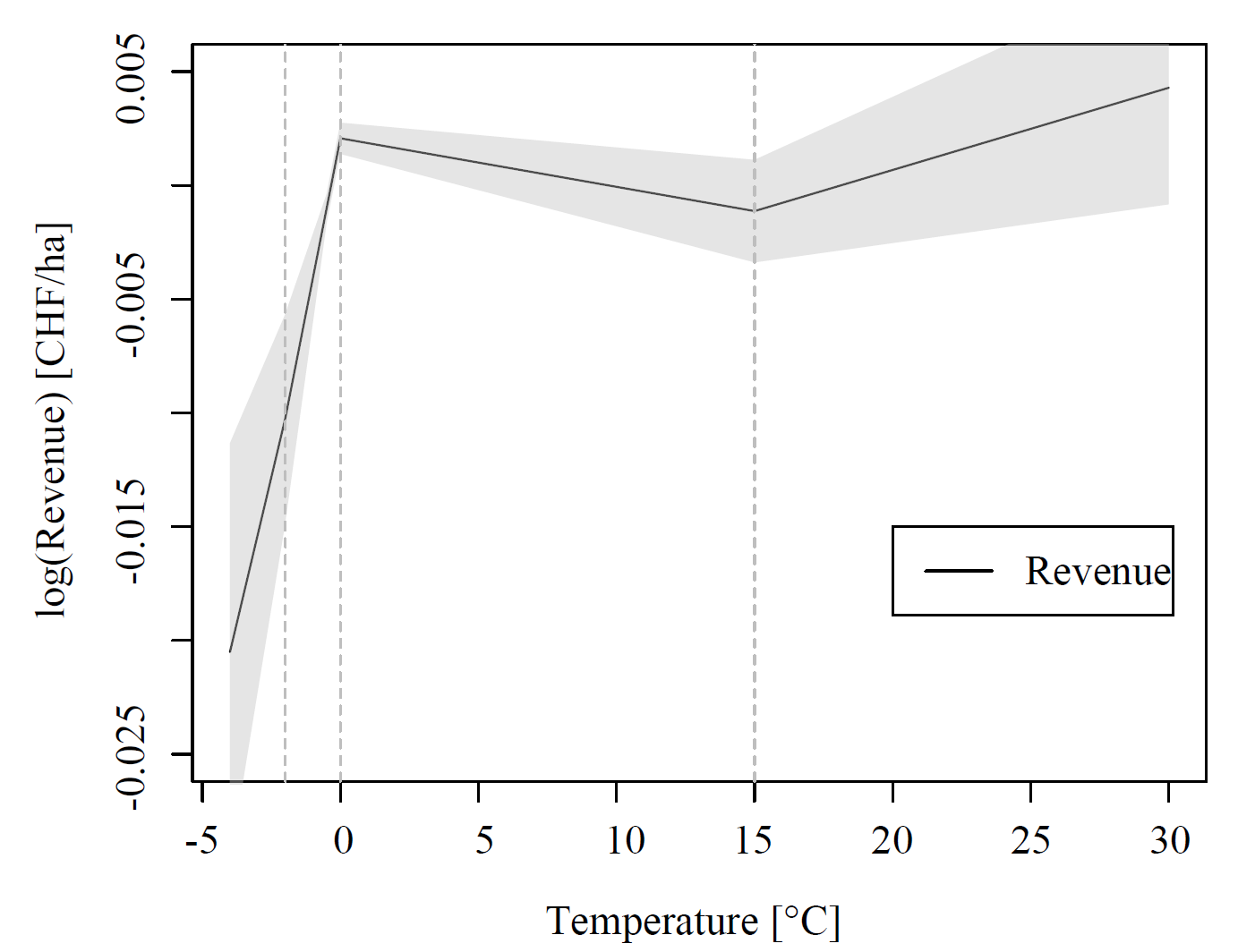


**Figure S1:**

Non-linear revenue (yield multiplied by price) response to a one hour temperature exposure at the respective x-axis temperature during apple flowering. Shaded areas represent 95% confidence intervals clustered by year and orchard. Dashed grey lines indicate interval breaks of the piecewise linear specification of the temperature impact.

Table S2: Full regression model results

|  |  | Dependent variable | | |
| --- | --- | --- | --- | --- |
|  |  | **Log Price**  **[CHF/kg]** | **Log Yield**  **[kg/ha]** | **Log Revenue**  **[CHF/ha]** |
|  |  |  |  |  |
| Temperature exposure < -2°C^1^ | $\beta_{1}$ | 0.106 (0.026) | -0.017 (0.014) | 0.123 (0.028) |
| Temperature exposure -2°C - 0 °C^1^ | $\beta_{2}$ | 0.130 (0.030) | 0.019 (0.015) | 0.145 (0.032) |
| Temperature exposure 0°C - 15 °C^1^ | $\beta_{3}$ | -0.005 (0.001) | -0.000 (0.001) | 0.000 (0.001) |
| Temperature exposure > 15 °C^1^ | $\beta_{4}$ | 0.008 (0.002) | 0.001 (0.001) | 0.009 (0.002) |
| Heat spells [days > 30°C] ^2^ | $\delta_{1}$ | 0.001 (0.004) | 0.001 (0.001) | 0.001 (0.000) |
| Chilling hours [0< h < 7.2°C] ^3^ | $\delta_{2}$ | -0.005 (0.003) | 0.005 (0.001) | -0.001 (0.003) |
| Orchard Fixed Effects | $\mu_{i}$ | Yes | Yes | Yes |
| Year Fixed Effects | $\upsilon_{t}$ | Yes | Yes | Yes |
| N |  | 2’385 | 2’441 | 2’385 |
| R^2^ |  | 0.52 | 0.64 | 0.50 |

Values in parentheses are cluster (by year and orchard) and heteroscedasticity robust standard errors.

^1^ Exposure for the piecewise linear response function considers only temperature during the blooming phase as measured by phenological phases. ‘*first flowers open’* to ‘e*nd of flowering: all petals fallen’*

^2^ Heat spells were count during the whole year

^3^ Chilling hours are count only during the winter months, i.e. during winter dormancy as measured by phenological phases ‘*end of leaf fall*’ and ‘*start of bud development*’


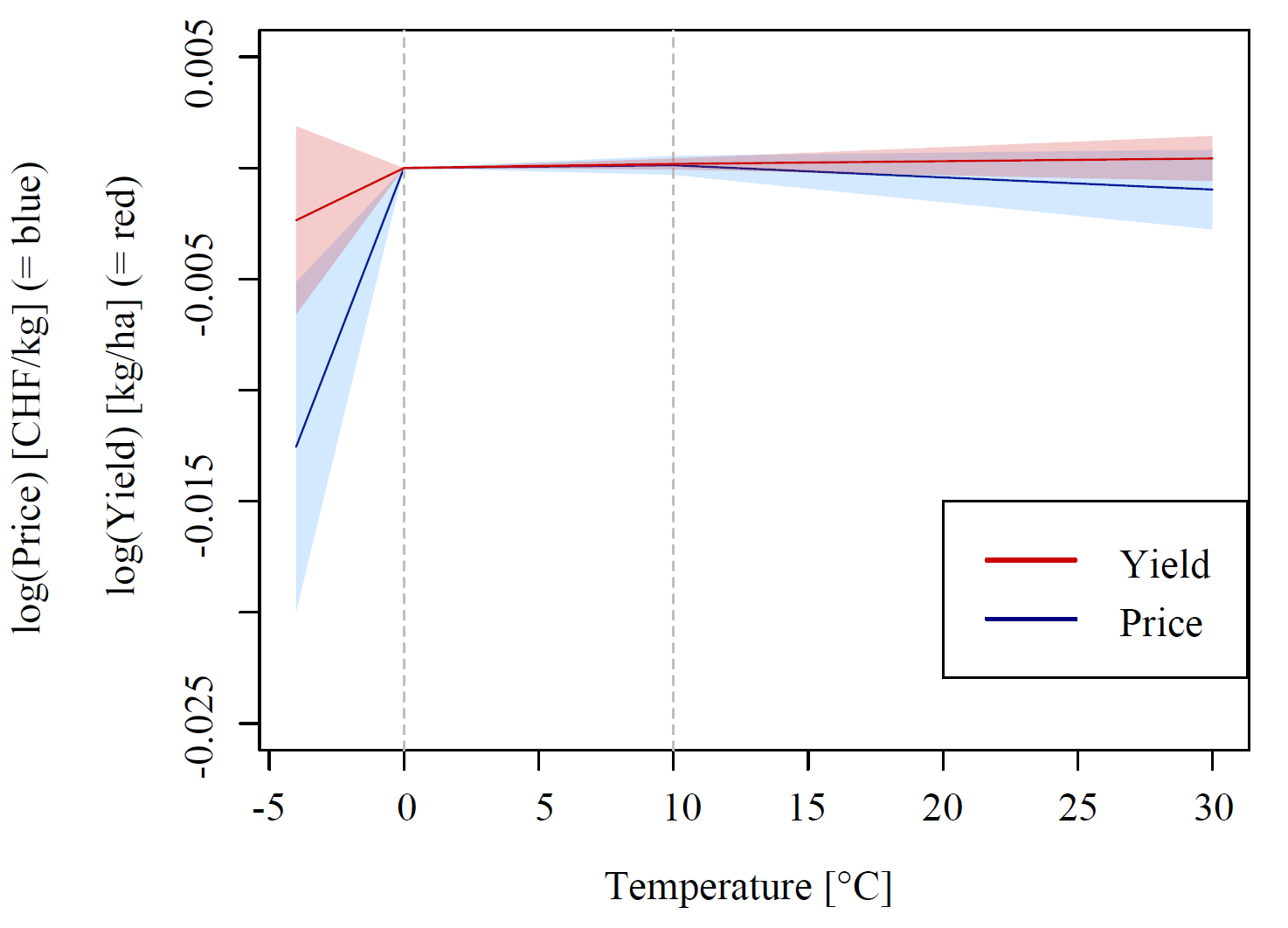


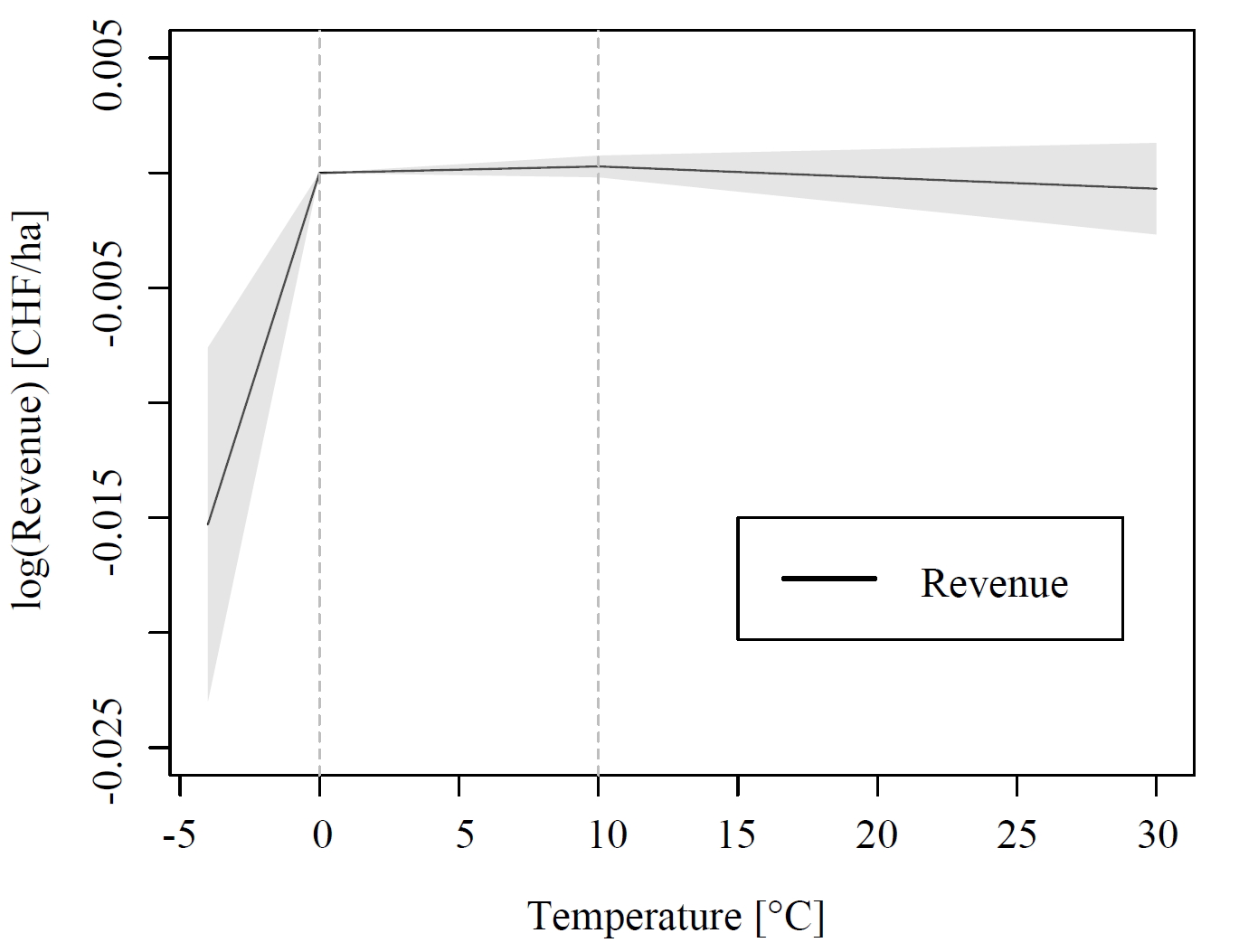


**Figure S2:**

Non-linear yield, price and revenue response to a one hour temperature exposure at the respective x-axis temperature during apple flowering. Effects are estimated for three temperature intervals (<0°C, 0-10°C,>10°C). Shaded areas represent 95% confidence intervals clustered by year and orchard. Dashed grey lines indicate interval breaks of the piecewise linear specification of the temperature impact.


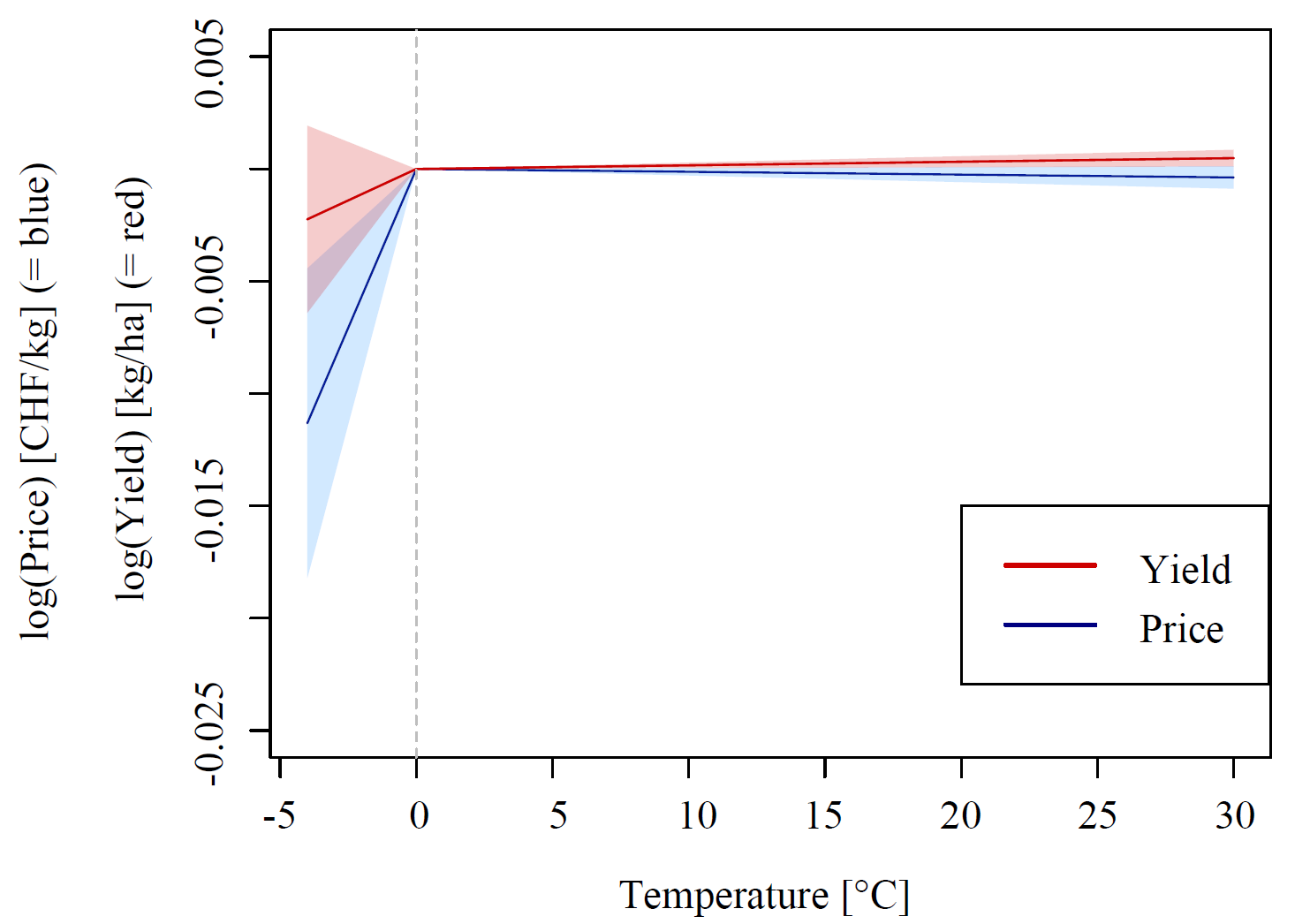


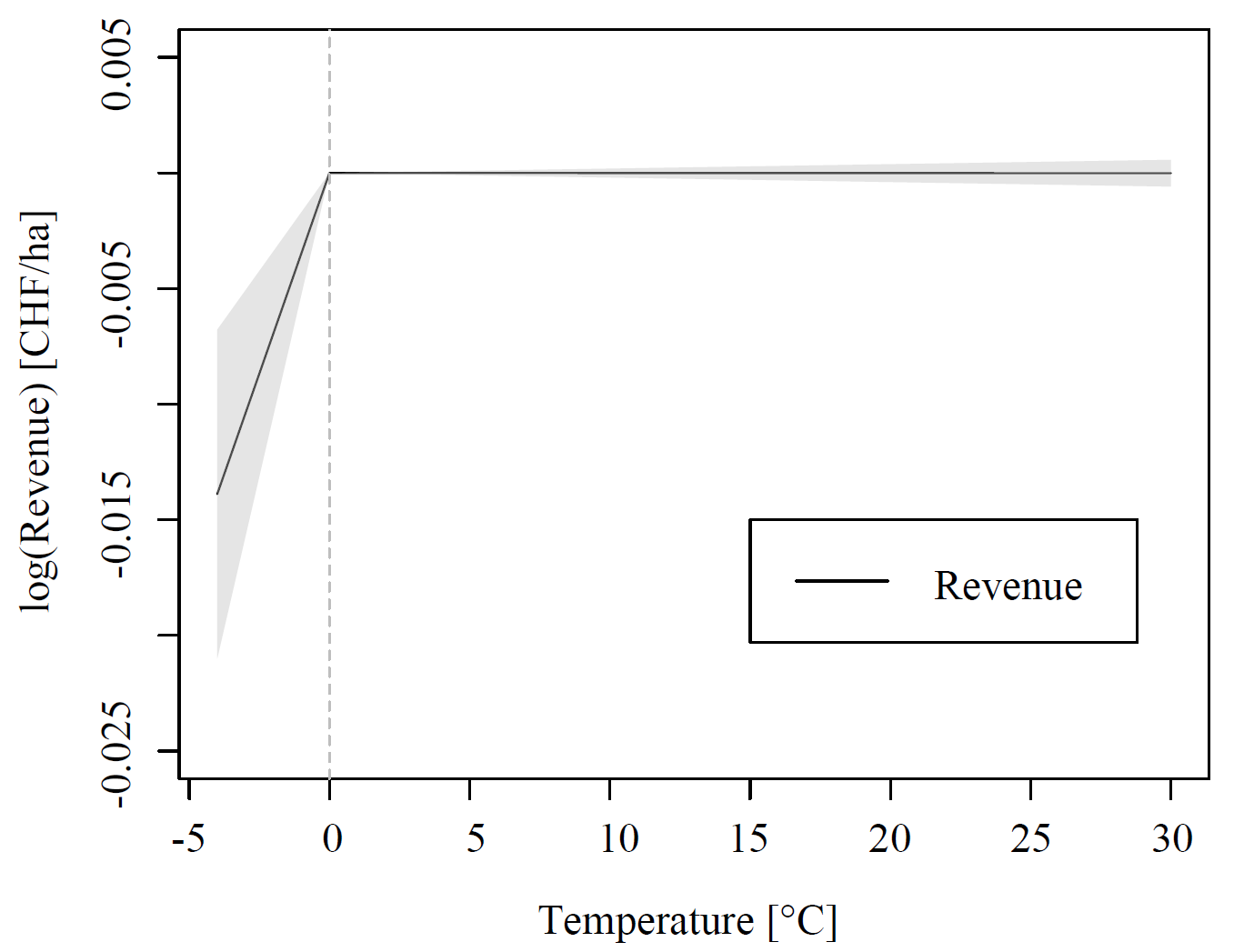


**Figure S3:**

Non-linear yield, price and revenue response to a one hour temperature exposure at the respective x-axis temperature during apple flowering. Effects are estimated for two temperature intervals (<0°C, >0°C). Shaded areas represent 95% confidence intervals clustered by year and orchard. Dashed grey lines indicate interval breaks of the piecewise linear specification of the temperature impact.


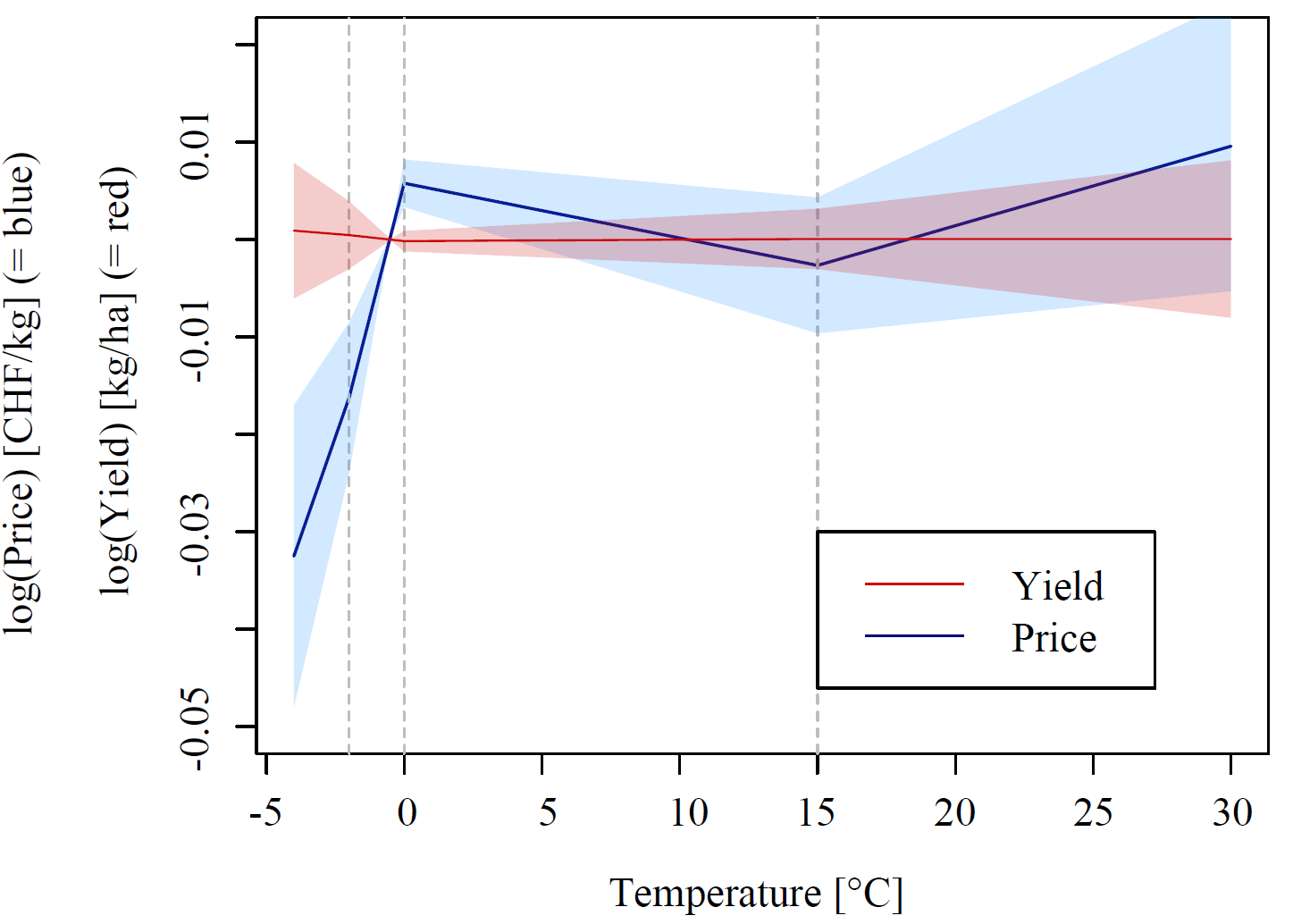

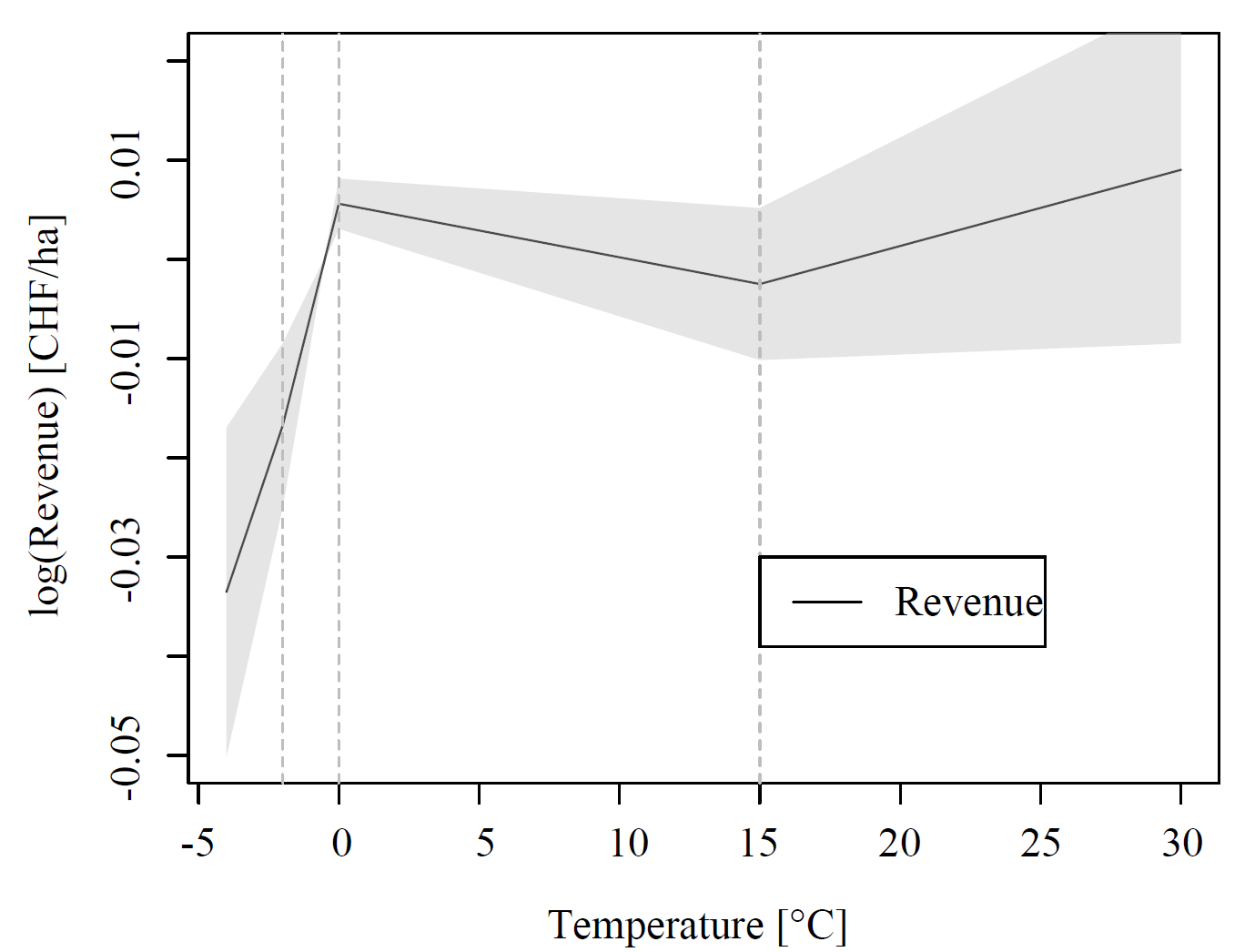


**Figure S4:**

Subsample cultivar *Golden Delicious* (N=379). Non-linear yield, price and revenue response to a one hour temperature exposure at the respective x-axis temperature during apple flowering. Shaded areas represent 95% confidence intervals clustered by year and orchard. Dashed grey lines indicate interval breaks of the piecewise linear specification of the temperature impact.


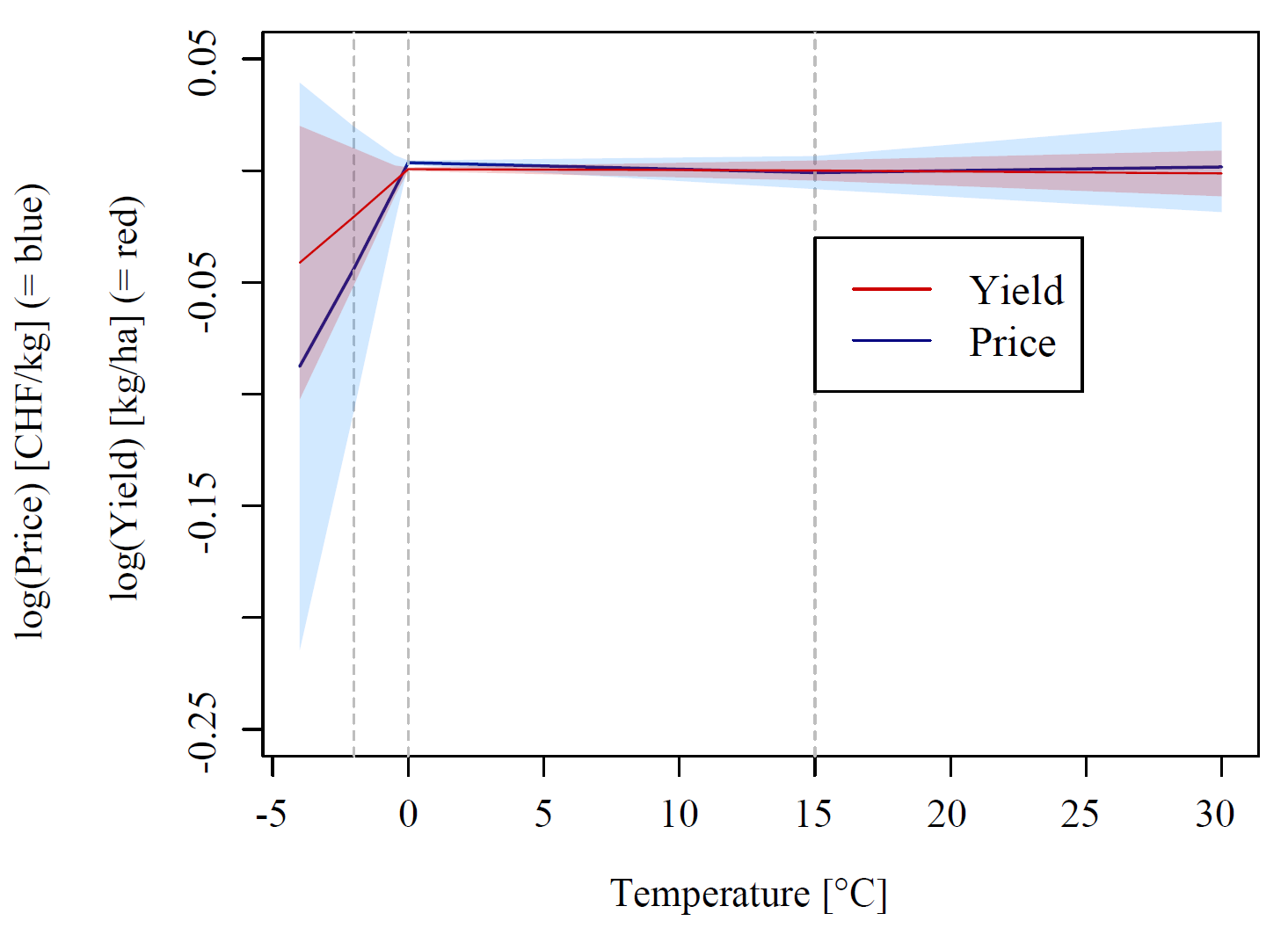


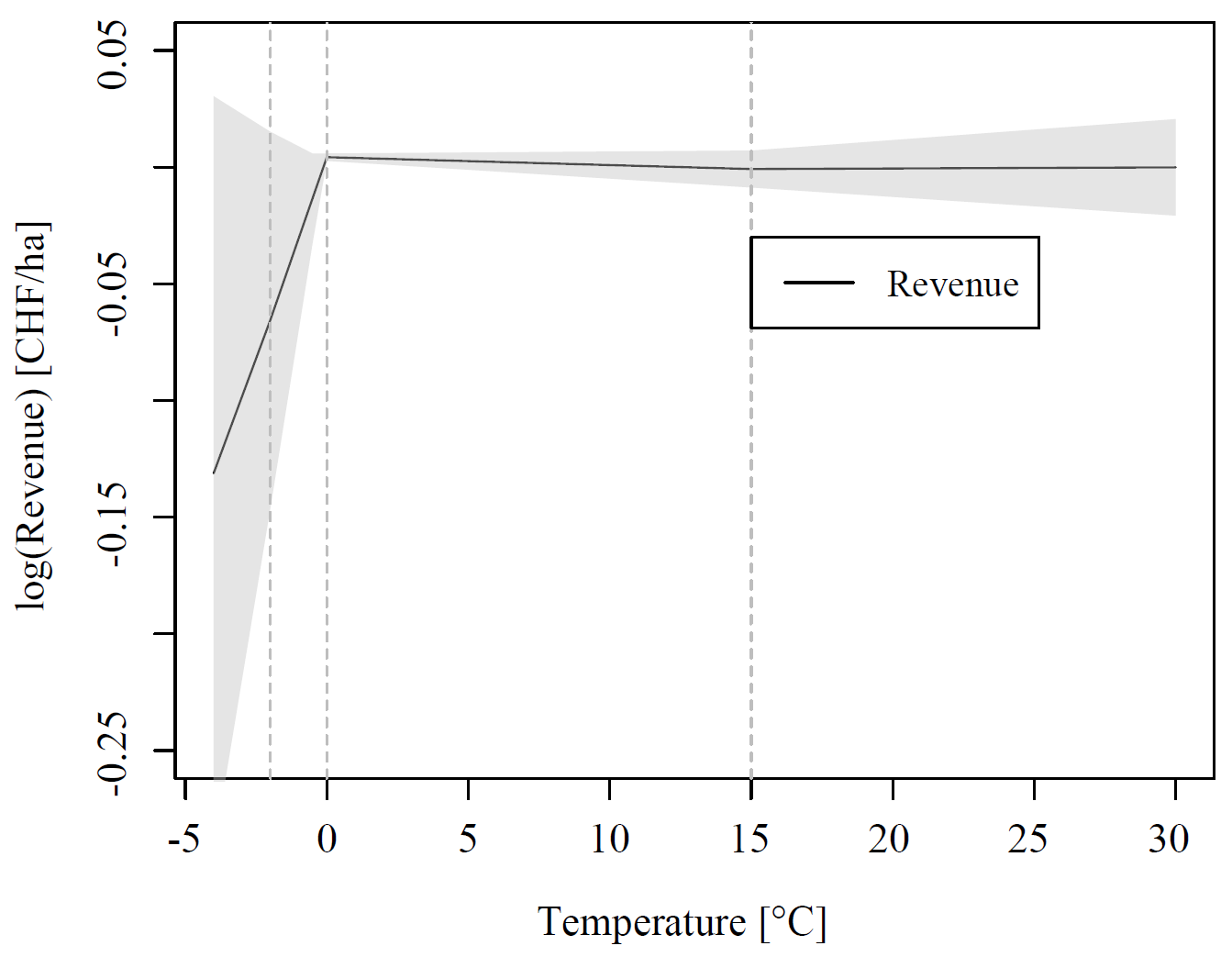


**Figure S5:**

Subsample cultivar *Gala* (N=230). Non-linear yield, price and revenue response to a one hour temperature exposure at the respective x-axis temperature during apple flowering. Shaded areas represent 95% confidence intervals clustered by year and orchard. Dashed grey lines indicate interval breaks of the piecewise linear specification of the temperature impact.


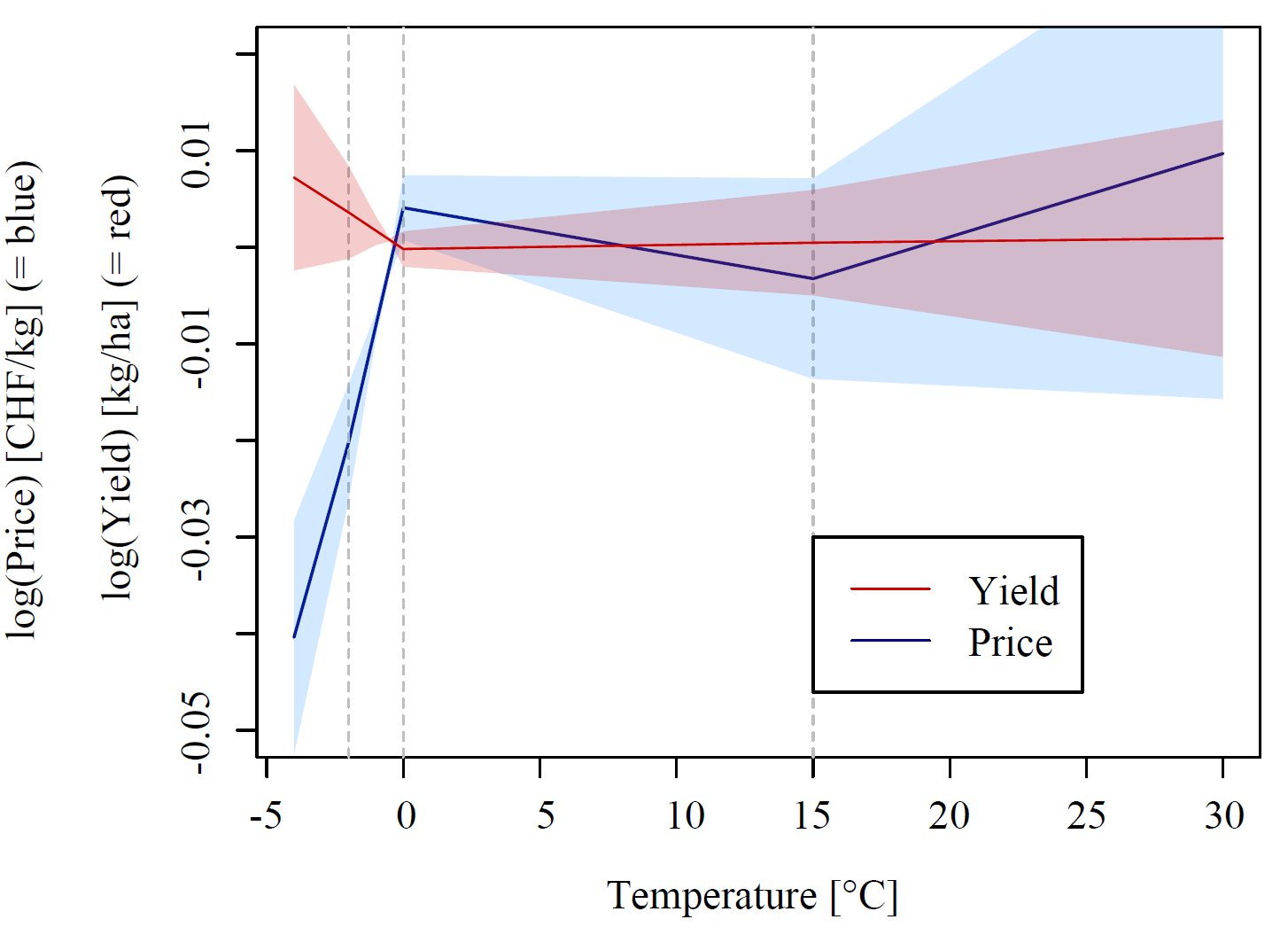


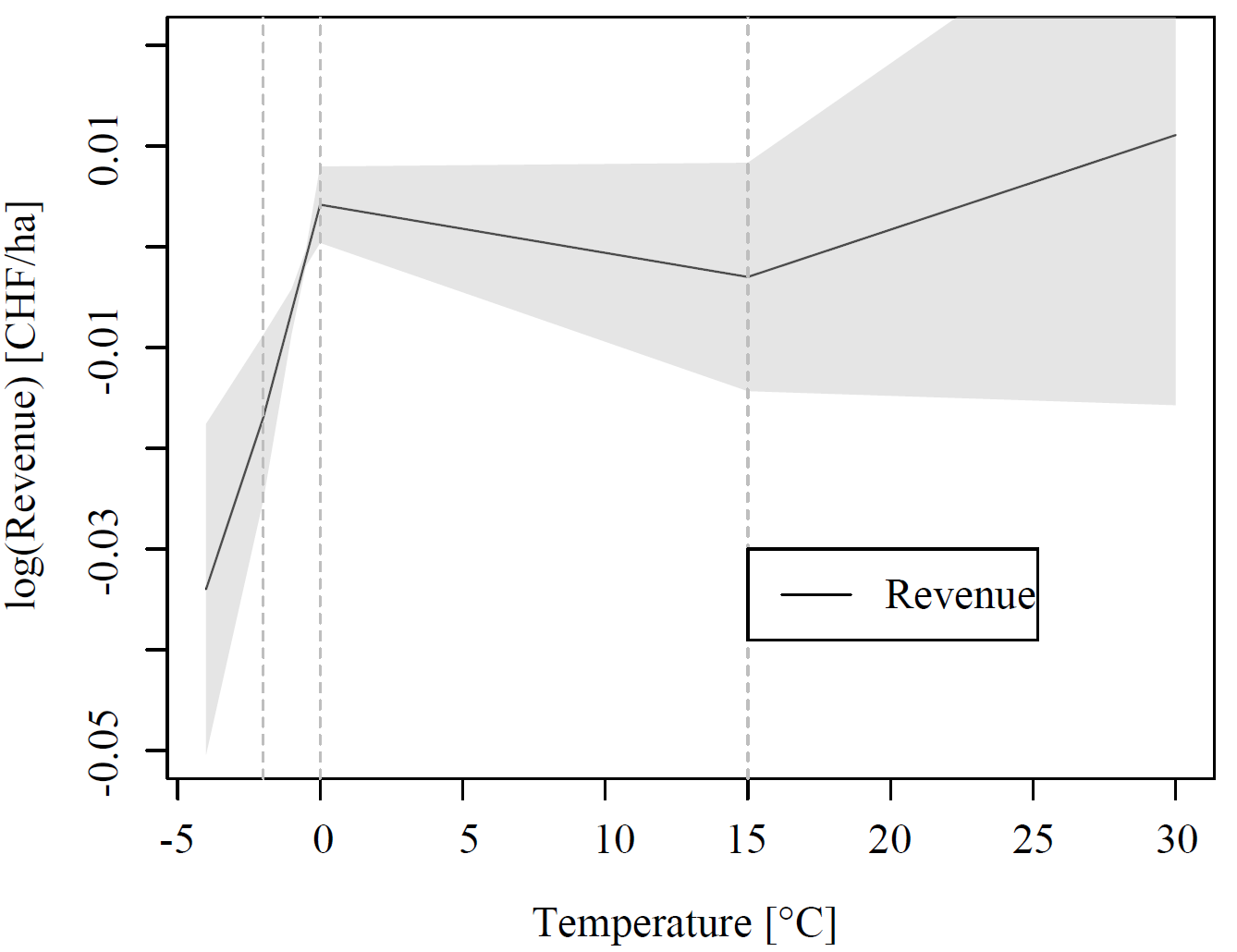


**Figure S6:**

Subsample cultivar *Jonagold* (N=184). Non-linear yield, price and revenue response to a one hour temperature exposure at the respective x-axis temperature during apple flowering. Shaded areas represent 95% confidence intervals clustered by year and orchard. Dashed grey lines indicate interval breaks of the piecewise linear specification of the temperature impact.


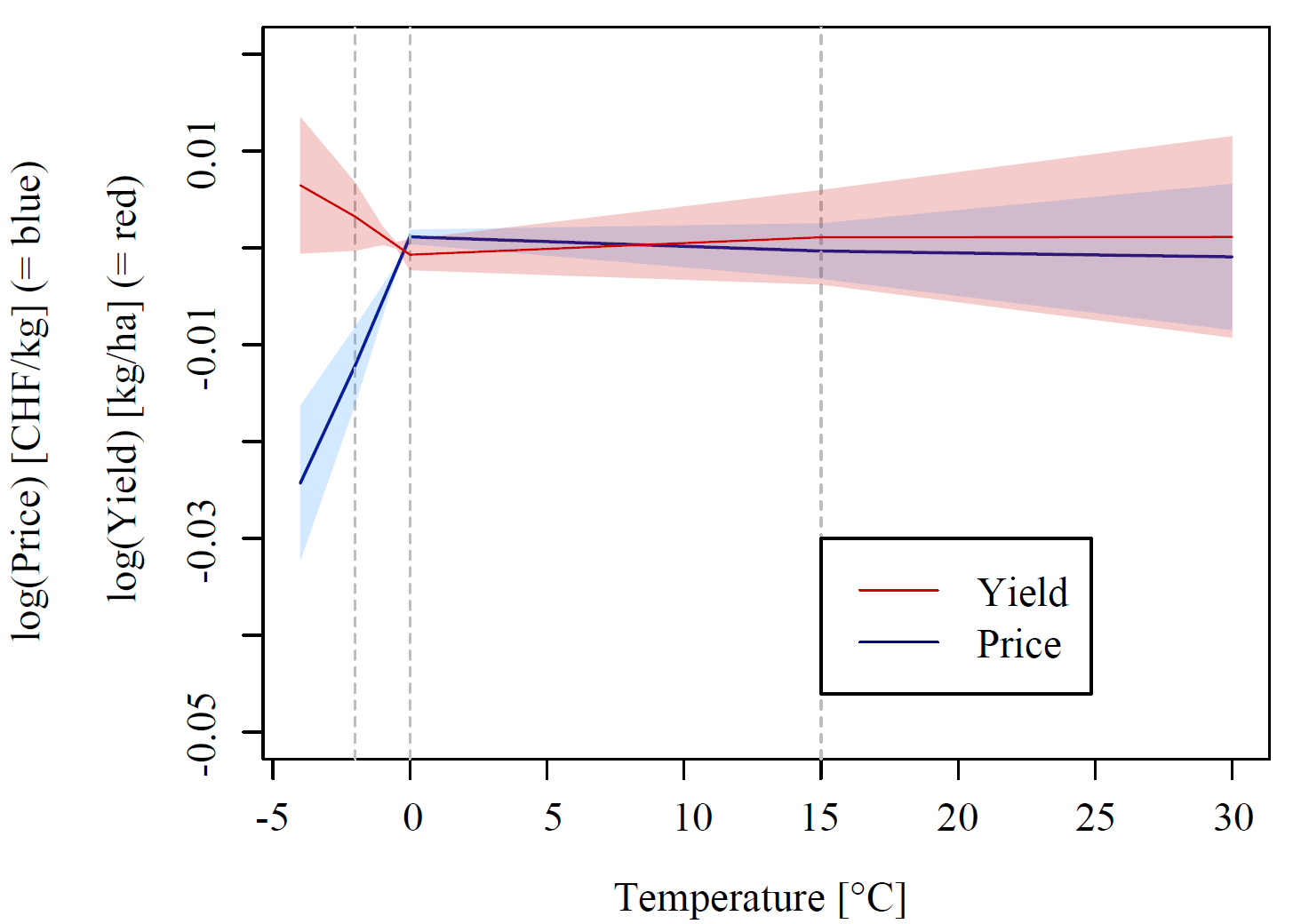


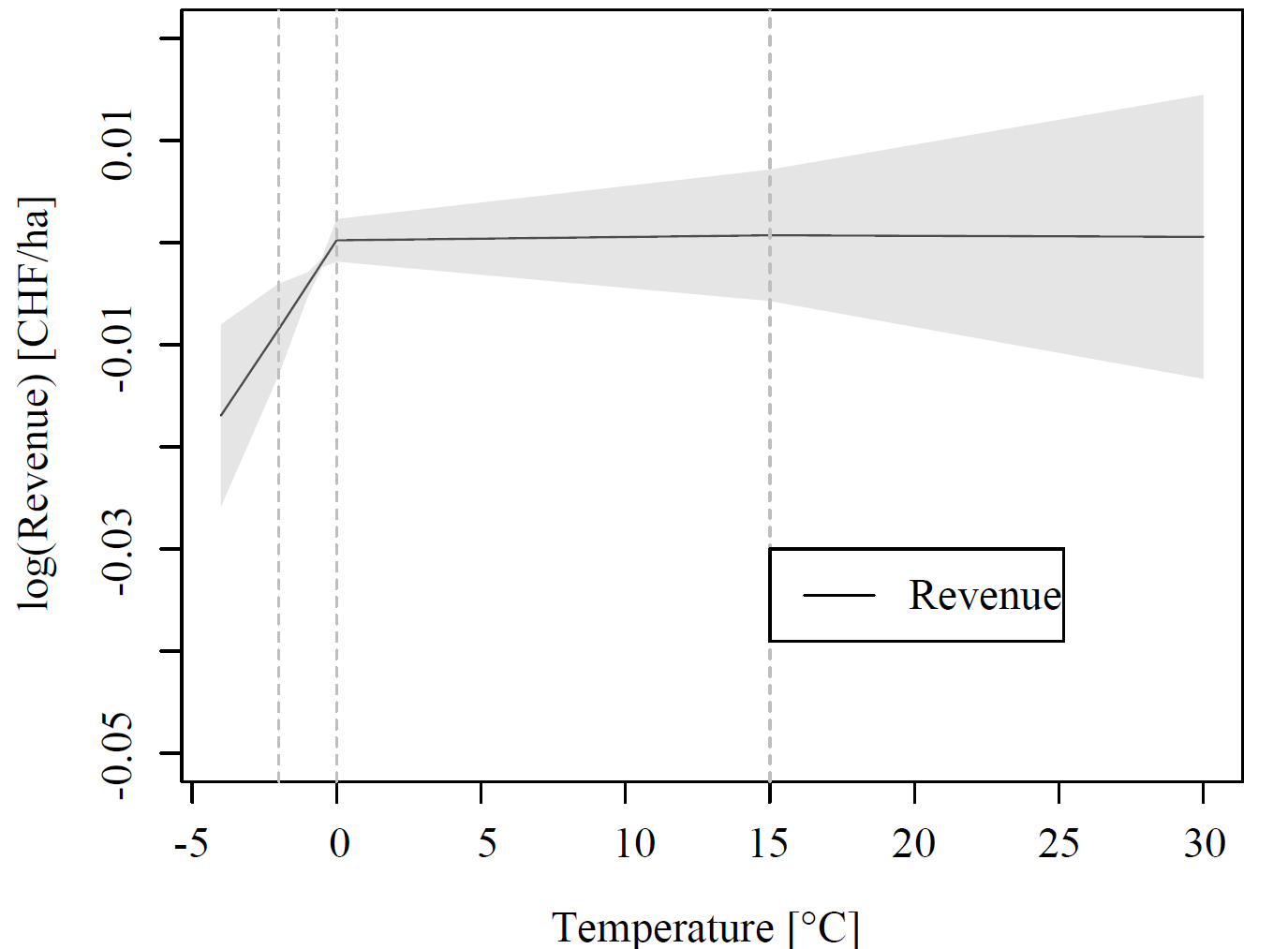


**Figure S7:**

Subsample cultivar *Maigold* (N=150). Non-linear yield, price and revenue response to a one hour temperature exposure at the respective x-axis temperature during apple flowering. Shaded areas represent 95% confidence intervals clustered by year and orchard. Dashed grey lines indicate interval breaks of the piecewise linear specification of the temperature impact.


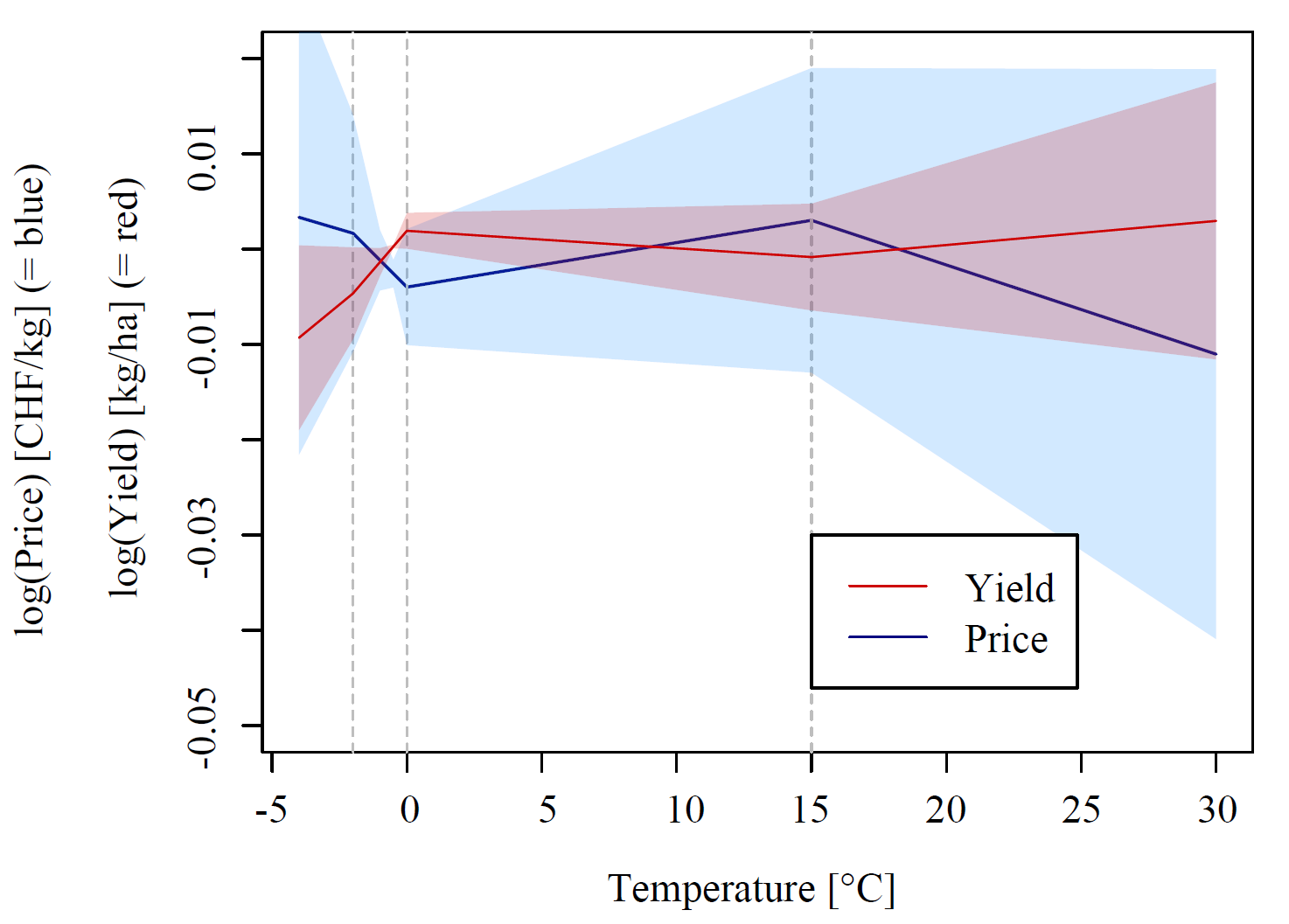


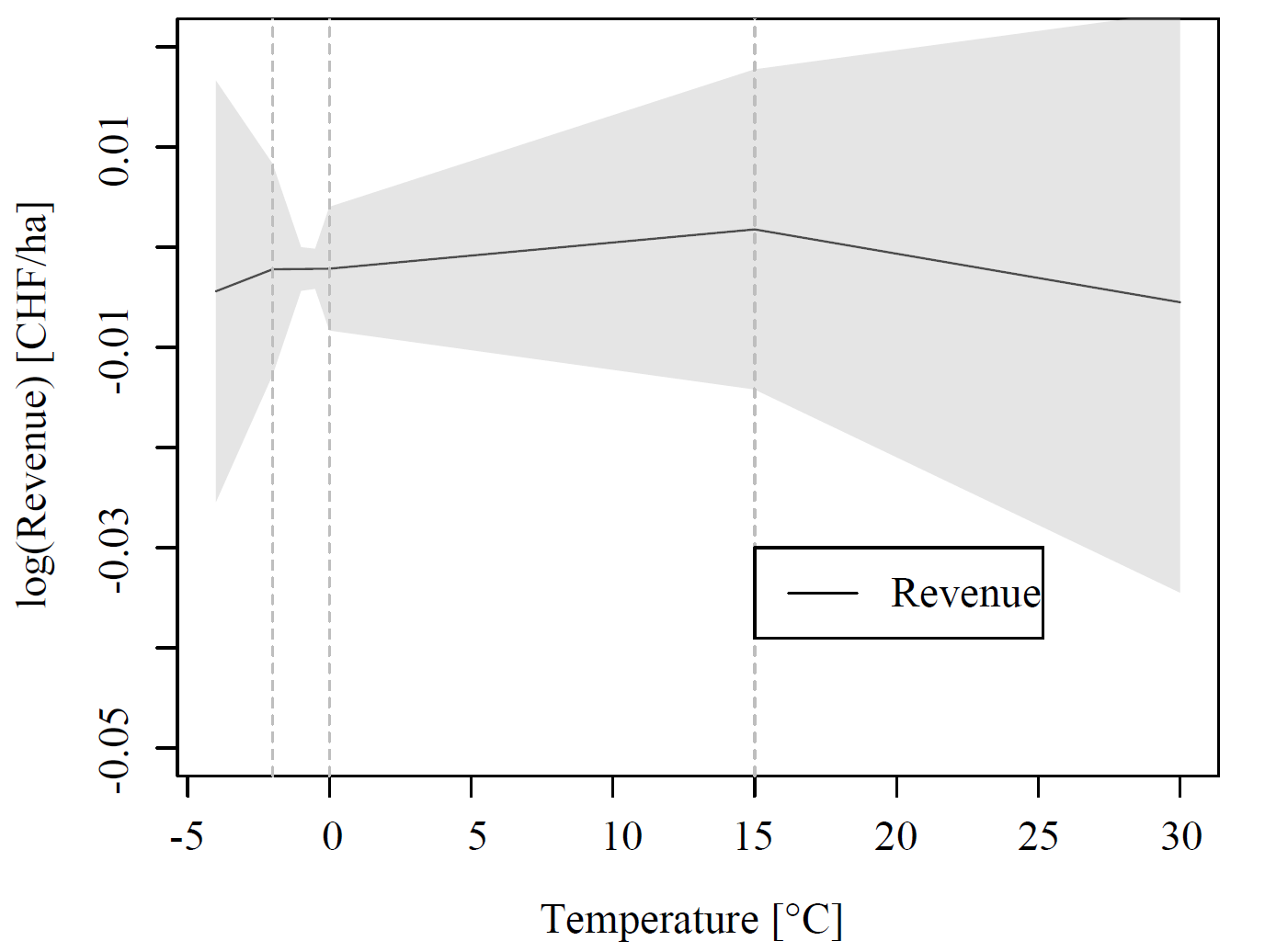


**Figure S8:**

Subsample ‘placebo’ cultivar *Boskoop* (N=142). Non-linear yield, price and revenue response to a one hour temperature exposure at the respective x-axis temperature during apple flowering. Shaded areas represent 95% confidence intervals clustered by year and orchard. Dashed grey lines indicate interval breaks of the piecewise linear specification of the temperature impact.


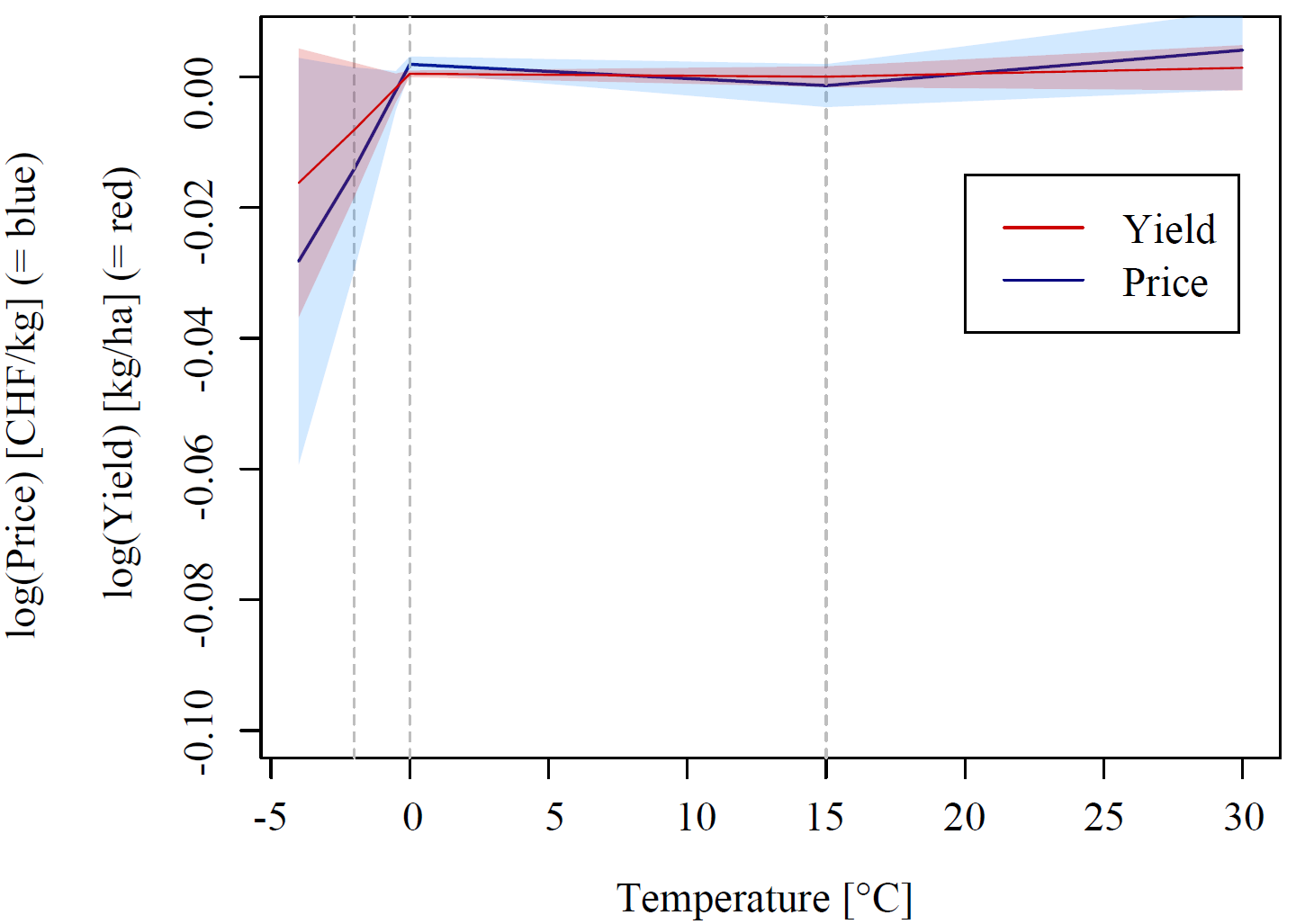


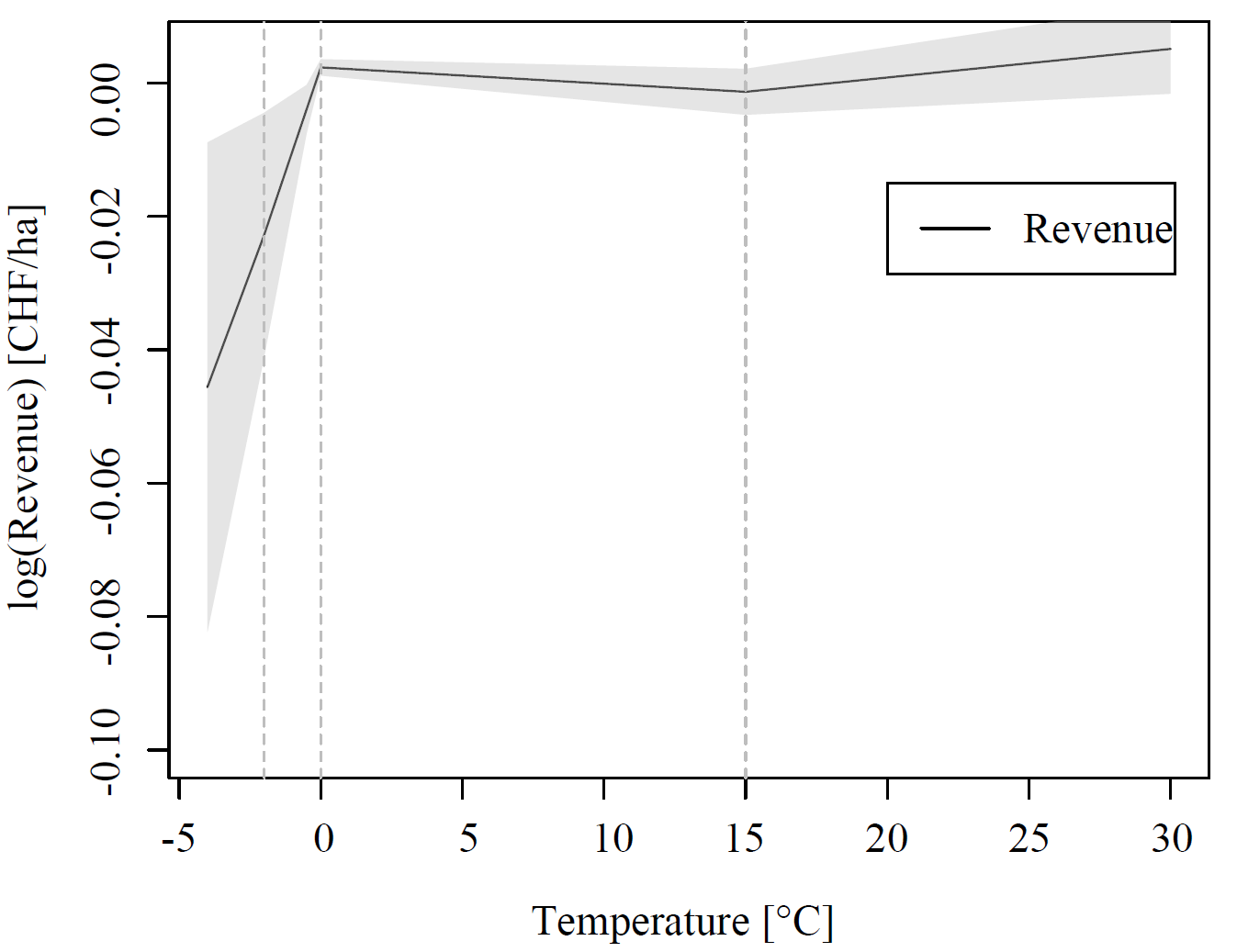


**Figure S9:**

Subsample using only data of the phenology matching steps 1 & 2 (N=2137). Non-linear yield, price and revenue response to a one hour temperature exposure at the respective x-axis temperature during apple flowering. Shaded areas represent 95% confidence intervals clustered by year and orchard. Dashed grey lines indicate interval breaks of the piecewise linear specification of the temperature impact.


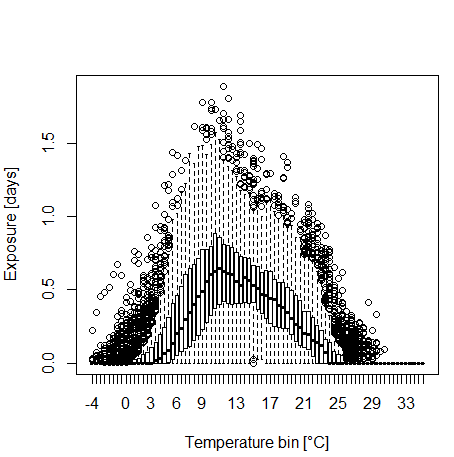


**Figure S10:** Temperature exposure during the flowering stage across 0.5°C temperature intervals.


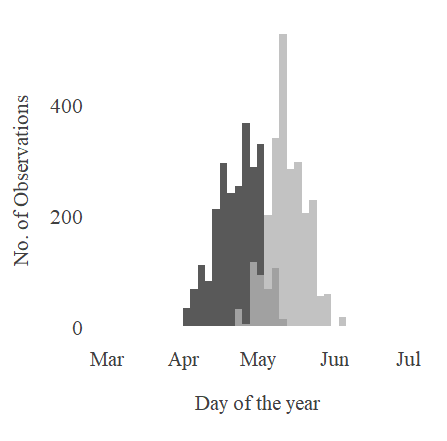


**Figure S11**: Start (dark grey) and end (light grey) dates of flowering

## References

[14] Vitasse, Y., & Rebetez, M. (2018). Unprecedented risk of spring frost damage in Switzerland and Germany in 2017. *Climatic Change*, *149*(2), 233-246.

[18] Rodrigo, J. (2000). Spring frosts in deciduous fruit trees—morphological damage and flower hardiness. *Scientia Horticulturae*, 85(3), 155-173.

[25] Blanke, M., & Kunz, A. (2010). Effects of climate change on pome fruit phenology and precipitation. *Acta Hort. (ISHS)* 922, 381-386.

[26] Eccel, E., Rea, R., Caffarra, A., & Crisci, A. (2009). Risk of spring frost to apple production under future climate scenarios: the role of phenological acclimation. *International journal of biometeorology*, *53*(3), 273-286.

[27] Blanke, M., & Kunz, A. (2009). Einfluss rezenter Klimaveränderungen auf die Phänologie bei Kernobst am Standort Klein-Altendorf–anhand 50-jähriger Aufzeichnungen. *Erwerbs-Obstbau*, *51*(3), 101-114.

[28] Stöckle, C. O., Nelson, R. L., Higgins, S., Brunner, J., Grove, G., Boydston, R. & Kruger, C. (2010). Assessment of climate change impact on Eastern Washington agriculture. *Climatic Change*, 102(1), 77-102.

[37] Racsko, J., & Schrader, L. E. (2012). Sunburn of apple fruit: Historical background, recent advances and future perspectives. *Critical reviews in plant sciences*, *31*(6), 455-504.

[38] Pérez-Jiménez, J., & Saura-Calixto, F. (2015). Macromolecular antioxidants or non-extractable polyphenols in fruit and vegetables: Intake in four European countries. *Food Research International*, *74*, 315-323.

[39] Fuhrer, J., Smith, P., & Gobiet, A. (2014). Implications of climate change scenarios for agriculture in alpine regions—A case study in the Swiss Rhone catchment. *Science of the Total Environment*, 493, 1232-1241.

[40] Hoffmann, H., & Rath, T. (2013). Future bloom and blossom frost risk for Malus domestica considering climate model and impact model uncertainties. *PloSone*, *8*(10), e75033.

[41] Garratt, M. P., Breeze, T. D., Jenner, N., Polce, C., Biesmeijer, J. C., & Potts, S. G. (2014). Avoiding a bad apple: Insect pollination enhances fruit quality and economic value. *Agriculture, ecosystems & environment*, *184*, 34-40.

[42] Menapace, L., Colson, G., & Raffaelli, R. (2013). Risk aversion, subjective beliefs, and farmer risk management strategies. *American Journal of Agricultural Economics*, 95(2), 384-389.

[43] Federal Office for Agriculture (2016). Obst- und Tafeltraubenanlagen der Schweiz 2015, Bern

[44] Finger, R. (2012). Effects of crop acreage and aggregation level on price-yield correlations. *Agricultural Finance Review*, *72*(3), 436-455.

[45] Foudi, S., & Erdlenbruch, K. (2011). The role of irrigation in farmers’ risk management strategies in France. *European Review of Agricultural Economics*, *39*(3), 439-457.

[46] Finger, R., & Lehmann, N. (2012). Policy reforms to promote efficient and sustainable water use in Swiss agriculture. *Water Policy*, *14*(5), 887-901.

[47] Snyder, R. L., & Melo-Abreu, J. P. (2005). Frost protection: fundamentals, practice and economics. Food and Agriculture Organization of the United Nations, Rome

[48] Dalhaus, T., & Finger, R. (2016). Can Gridded Precipitation Data and Phenological Observations Reduce Basis Risk of Weather Index–Based Insurance?. *Weather, Climate, and Society*, 8(4), 409-419.

[49] Dalhaus, T., Musshoff, O., & Finger, R. (2018). Phenology Information Contributes to Reduce Temporal Basis Risk in Agricultural Weather Index Insurance. *Scientific reports*, *8*(1), 46.

1. Organic orchards are slightly overrepresented in our sample. [↑](#footnote-ref-1)
